# Modeling community COVID-19 transmission risk associated with U.S. universities

**DOI:** 10.1038/s41598-023-28212-z

**Published:** 2023-01-25

**Authors:** J. A. Uelmen, H. Kopsco, J. Mori, W. M. Brown, R. L. Smith

**Affiliations:** 1 Department of Epidemiology, School of Public Health, University of Michigan, Ann Arbor, USA; 2 Department of Pathobiology, School of Veterinary Medicine, University of Illinois, Champaign, USA; 3 Natural Resources and Environmental Sciences, University of Illinois, Champaign, USA

**Keywords:** Infectious diseases, Viral infection, Risk factors

## Abstract

The ongoing COVID-19 pandemic is among the worst in recent history, resulting in excess of 520,000,000 cases and 6,200,000 deaths worldwide. The United States (U.S.) has recently surpassed 1,000,000 deaths. Individuals who are elderly and/or immunocompromised are the most susceptible to serious sequelae. Rising sentiment often implicates younger, less-vulnerable populations as primary introducers of COVID-19 to communities, particularly around colleges and universities. Adjusting for more than 32 key socio-demographic, economic, and epidemiologic variables, we (1) implemented regressions to determine the overall community-level, age-adjusted COVID-19 case and mortality rate within each American county, and (2) performed a subgroup analysis among a sample of U.S. colleges and universities to identify any significant preliminary mitigation measures implemented during the fall 2020 semester. From January 1, 2020 through March 31, 2021, a total of 22,385,335 cases and 374,130 deaths were reported to the CDC. Overall, counties with increasing numbers of university enrollment showed significantly lower case rates and marginal decreases in mortality rates. County-level population demographics, and not university level mitigation measures, were the most significant predictor of adjusted COVID-19 case rates. Contrary to common sentiment, our findings demonstrate that counties with high university enrollments may be more adherent to public safety measures and vaccinations, likely contributing to safer communities.

## Introduction

In the United States (U.S.), early assessments measuring the impact of SARS-CoV-2 (COVID-19) transmission on college campuses and their surrounding communities were challenging due to heterogeneous mitigation and testing strategies. Many public health experts warned during the summer of 2020 that reopening campuses before vaccines were widely available was risky, and subsequent outbreaks among students, staff, and faculty within weeks of arriving on campus for the Fall semester quickly led to shutdowns^1–3^.

Due to the wide variation in the mitigation and containment strategies implemented during the Fall 2020 semester on college campuses across the U.S.^3^, questions remain about which strategies were most effective in lowering incidence of COVID-19 on campuses, and how campuses impacted the surrounding non-university communities. The overall sentiment, both in public opinion and some modeling studies, has been that college campuses fuel outbreaks and spread into the local community^4,5^. One study found that re-opening campuses to in-person instruction contributed to increased human mobility and was strongly associated with increased COVID-19 incidence in neighboring communities^6^. However, this study only analyzed teaching modality (e.g., in-person vs. hybrid vs. fully online courses) and did not examine other mitigation and containment approaches that were implemented. Stratifying the analysis by collective COVID-response plan may reveal more information about the impact of these measures. In a study of a large (~ 40,000 individuals), northeastern urban university with ample mitigation strategies (e.g. rapid delivery of SARS-CoV-2 polymerase chain reaction (PCR) test results, contact tracing, face mask use, reduced class size and density, and enhancement of all building air systems) more than half of cases were traced to a non-campus source, and no cases were attributed to classroom exposure^7^. These contradictions spawn a twofold question: are universities, particularly those with large (> 15,000) student populations, fueling community spread of COVID-19, and how?

Observations of COVID-19 responses in the U.S. and other countries, and models of potential outcomes for the Fall semester demonstrated that a safe re-opening of university campuses depended on rigid adherence to containment (e.g., testing, isolation, contact tracing, and quarantine) and mitigation strategies (e.g., masking, ventilation, and social distancing). A key component of this approach was frequent testing of students, faculty, and staff for COVID-19^1,8,9^. A stochastic model by Gressman and Peck^10^ evaluating mitigation found that, in addition to high COVID-19 test specificity, remote instruction and a high capacity for student quarantine were critical to successfully managing outbreaks. Findings from an agent-based model also suggested that reducing direct contact in residence halls and classrooms through class size caps or hybrid options, combined with masking and social distancing, could keep the campus viral reproductive rate at or below 1.0^11^. Additionally, evidence suggested that frequent testing and strict social distancing measures may prevent university shutdowns and the economic penalties associated with such a disruption, potentially outweighing the costs of implementing such surveillance^12^. However, given that providing masks, implementing remote learning, and conducting regular testing requires up front and ongoing funding that not all colleges and universities have, modification of these mitigation strategies is often needed ^13^.

The aims of this investigation were twofold: (1) to estimate U.S. county-level COVID-19 case and mortality rates and compare those of counties with and without college campuses, and (2) to model the impact of mitigation efforts on case rates of COVID-19 in a sample of U.S. counties with college campuses. We hypothesized that colleges and universities with robust COVID-19 response plans (e.g. those that have increasing adherence to mask wearing, social distancing, vaccination rates, frequent testing, education campaigns, etc.) would have a protective effect both on campus members and their surrounding communities, and that overall, presence of universities would not negatively impact the overall surrounding community.

## Methods

### Data sources

#### Cases and mortalities

SARS-CoV-2 laboratory-confirmed case and mortality data were acquired from the Centers for Disease Control and Prevention (CDC) data archive^14^. We selected the time period of January 1, 2020 to March 30, 2021 to answer question one for three reasons: (1) the Spring (approximately January–May) and Fall (approximately September-December) periods capture the entirety of a typical university calendar year, providing a control period to adequately measure COVID-19 transmission in local communities, (2) The commencement of the fall semester (September 1, 2020) is a critical point as it is approximately the median time point for this analysis, and (3) The U.S. experienced three distinctive COVID-19 “waves” over this period, creating a large representative dataset for comparing counties with campuses across the country.

Each case in this dataset contained 32 elements, including demographics (e.g., age, race and ethnicity, and sex) and county/state of residence. Numerous reporting agencies provide this information to the CDC and as a result, lineage-specific COVID-19 designation, symptom onset, and/or test positive dates were often incomplete, inconsistent, and/or provisional. All cases were aggregated with no sequencing-specific lineage designation. If multiple dates were listed, a single case-positive date was selected from the following (using the same order of preference as the CDC) data: symptom onset date, test positive date, and CDC report date. If no date or county of residence information was provided, the case was excluded. All personally identifiable information (names, addresses, etc.) were removed by the CDC prior to public release of the data. Geographic locations for each case were aggregated to county-level for additional privacy protections.

#### University enrollment

University enrollment (UE) data were acquired from the Integrated Postsecondary Education Data System (IPEDS)^15^. Total enrollment by postsecondary institution for the Fall semester of 2018–2019 academic year was aggregated by county and divided into four categories. Counties with total enrollment x $\ge$ 15,000, 15,000 > x > 5000, 5000 $\ge$ x > 0, or no enrollment, were labeled large (n = 253), medium (n = 361), small (n = 792), and absent (n = 1641), respectively. These cut points were selected based on natural breaks (Jenks optimization method) in the numerically-aligned data.

#### U.S. population and other key covariates

County population was acquired from the American Community Survey (ACS) 2019 5-year estimate^16^. These data provide estimates of population by age from 60 months of sampling between January 1, 2015 and December 31, 2019. For purposes of this study, age groups have been categorized by 10-year intervals as 0–9, 10–19, 20–29, 30–39, 40–49, 50–59, 60–69, 70–79, and 80+ .

Other factors of interest in evaluating COVID-19 among populations at the county level included median household income^16^, unemployment^17^, COVID-19 community vulnerability index (CCVI)^18^, percentage of population that was vaccinated as of March 30, 2021 (at least 1 dose)^19^, and percentage vote by party candidate in the 2020 U.S. Presidential Election^20^.

#### University COVID-19 mitigation and containment policies

Highly incongruous COVID-19 policies were implemented across U.S. universities during of the Fall 2020 semester reopening. In light of this, to address Aim 2 we selected aggregated variables collected by the College Crisis Initiative at Davidson College that could be compared widely across a sample of institutions of various size and^21^. Only data from four-year universities were included in the analysis, and schools were organized by the enrollment size categories used in Aim 1. Reopening plan variables included the “Mode of Instruction” (MOI) as of September 1, 2020 and proposed on-campus COVID-19 testing strategy. Other institution factors (e.g. land grant university status and degree of campus urbanization)^15^ were included in the analysis due to potential impact of funding mechanisms and population density structures. Additionally, we evaluated county-aggregate factors such as self-reported masking adherence, state-instituted mask mandates^22^, median household income^16^ and unemployment rates^17^.

#### Statistical analysis

Aim 1: Cases and deaths were age-adjusted using the 2019 ACS 5-year estimates as the reference population. Each county’s COVID-19-confirmed cases and deaths were organized by age group for the time period between January 1, 2020 and March 30, 2021. These were then aggregated as a total for each outcome and age-adjusted.

Age-adjusted COVID-19 case and death rates were evaluated across three time scenarios: (1) January 1, 2020 to March 30, 2021; (2) By each “wave” period, determined by natural breaks between peaks among national cases (Wave 1: January 1, 2020–June 7, 2020, Wave 2: June 8, 2020–September 6, 2020, Wave 3: September 7, 2020–March 30, 2021); and (3) Before and during the Fall 2020 academic semester (January 1, 2020–August 31, 2020 and September 1, 2020–March 30, 2021)^23–25^.

All statistical comparisons of groups for Aim 1 were assessed using JMP® (Version 16.0)^26^. Group means were compared using Student’s t-test or Dunnett’s Test (control group = counties without university enrollment). Comparisons were significant at *p* < 0.05. Age-adjusted case and mortality rates were modeled separately as dependent variables. Models were stratified by county university enrollment size, totaling eight standard least squares regression models. Final model selections were made using backwards Bayesian Information Criterion (BIC)-based stepwise regression. Overall significance of an independent variable was assessed by its frequency of inclusion in each of the four county university enrollment types, as well as averaging the logWorth (calculated as − log_10_(*p* value)) of that variable across all four university county types.

Aim 2: Since the college reopening dataset contained both quantitative and qualitative variables, a factor analysis of mixed data using the *FactomineR* package^27^ in R (version 4.1.2) was conducted to identify important variables related to university reopening plans. Once the contributions of these variables to the overall variance of the dataset were assessed, a hierarchical cluster analysis was performed (using Bartlett test of sphericity, *p* < 0.05) to identify similar school mitigation strategy clusters associated with population-adjusted COVID-19 cases at the county level.

Finally, due to overdispersion in the case data, we fit negative binomial models using the *MASS* package^28^ to identify university COVID-19 mitigation strategies and other county-level variables that significantly predicted population-adjusted county COVID cases in the Fall 2020 semester. Model fit was evaluated with the *performance* package^29^ which gives a performance score based on the model’s BIC, Nagelkerke’s R^2^, and root-mean square error.

## Results

### Overall impact of universities on county-level COVID-19 cases and deaths (Aim 1)

**Figure 1:**
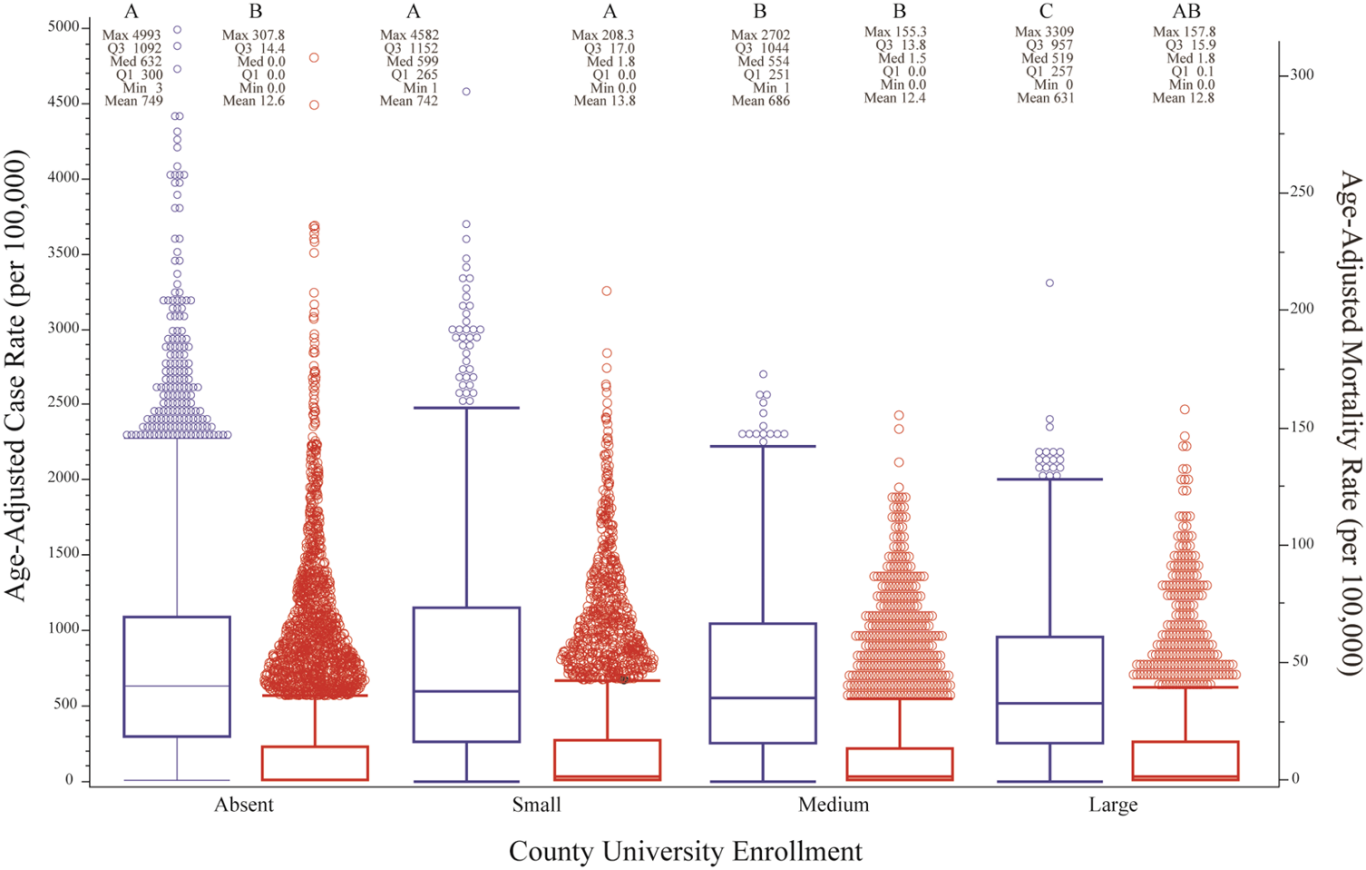
Box plot of laboratory-confirmed mean SARS-CoV-2 age-adjusted case (blue boxes) and mortality (red boxes) rates (per 100,000) by county university enrollment (none, small, medium, and large) among the United States population, from January 1, 2020 through March 30, 2021. Unique letters, arranged in descending order (highest mean value = A and so on), indicate statistically different groups (case and mortality rates, respectively; assessed by Student’s t-test, *p* value $\le$ 0.05). Individual circles represent outlier counties.

**Figure 2:**
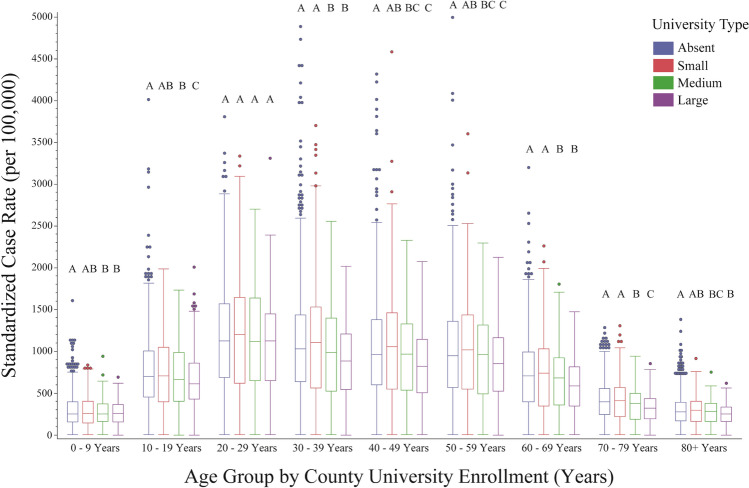
Box plot breakdown of laboratory-confirmed mean SARS-CoV-2 age-adjusted case rates (per 100,000) by age-group (binned by 10-year intervals). Unique letters, arranged in descending order (highest mean value = A and so on), indicate statistically different groups, assessed by Student’s t-test (*p* value $\le$ 0.05). Individual circles represent outlier counties.

**Figure 3:**
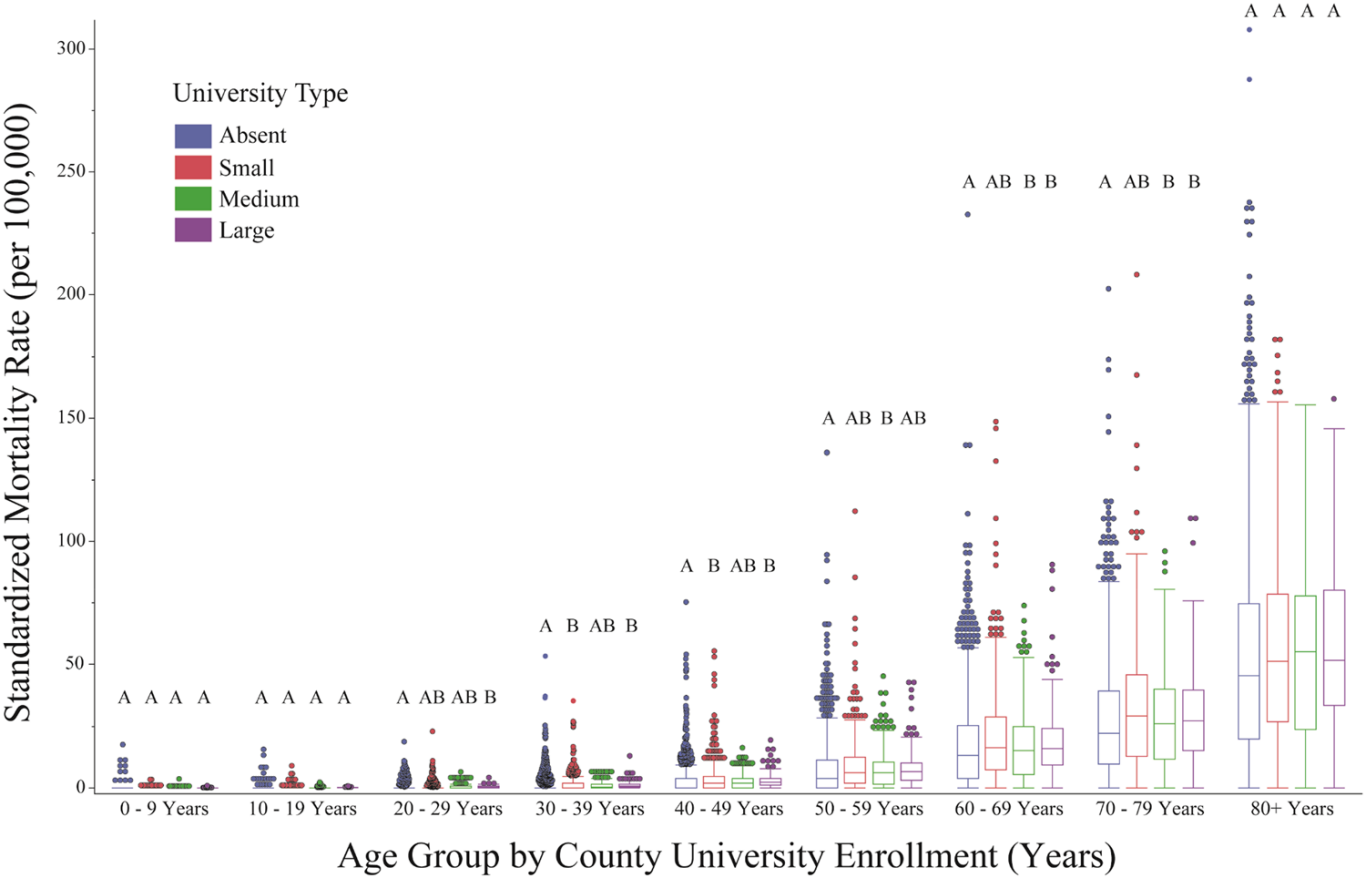
Box down breakdown of laboratory-confirmed mean SARS-CoV-2 age-adjusted mortality rates (per 100,000) by age-group (binned by 10-year intervals). Unique letters, arranged in descending order (highest mean value = A and so on), indicate statistically different groups, assessed by Student’s t-test (*p* value $\le$ 0.05). Individual circles represent outlier counties.

By March 30, 2021 a total of 22,385,335 cases and 374,130 deaths were reported to the CDC. Between January 1, 2020 and March 30, 2021, increasing county university enrollment was associated with significant reductions in COVID-19 case rates, but only slight differences in mortality rates (Table 1, Fig. 1). Compared to counties with no universities, this equated to a 1% reduction in cases among counties with small university enrollments, an 8% reduction among counties with medium university enrollments, and a 16% reduction among counties with large university enrollments. A comparison of standardized case and mortality rates by age group showed similar patterns across all enrollment types: 20–59-year-olds accounted for most cases, with the highest case rate in the 20–29-year-old age group (Fig. 2). Despite having a lower case rate than adults under 50, adults over 50 experienced the highest mortality rates regardless of county university enrollment type, and this rate increased with age (Fig. 3). On average, those > 80 had nearly twice the risk of death from COVID-19 as 70–79-year-olds, 2.5-times the risk as 60–69-year-olds, and 5-times the risk as 50–59-year-olds.

**Table 1:** Summary of United States population (U.S. Census 2010) and key socioeconomic, demographic, and political variables by race & ethnicity and county total university enrollment (none, small, medium, and large) (Aim 1). Cases and deaths labeled as unknown/missing denote individuals where gender was not provided.

Variable	County university enrollment			
	None (n = 1641, ref)	Small (n = 792)	Medium (n = 361)	Large (n = 253)
Population	33,260,133	49,051,160	56,626,128	184,344,451
	1	1.47**	1.70***	5.54***
**Socio-demographic descriptors**				
Average household income	$53,582	$55,475	$60,190	$67,690
	1	1.04**	1.12***	1.26***
Social vulnerability	0.45	0.54	0.56	0.60
	1	1.20***	1.24***	1.33***
% Unemployed	4	4.10	3.89	3.59
	1	1.03	0.97	0.90***
**2020 Presidential election**				
% Biden	0.28	0.32	0.38	0.51
	1	1.14***	1.36***	1.82***
% Trump	0.70	0.63	0.56	0.41
	1	0.90***	0.80***	0.59***
**Cases**				
Female	1,053,896	1,548,981	1,896,893	5,654,215
	1	1.47*	1.80***	5.37***
Male	957,526	1,391,310	1,701,010	5,125,302
	1	1.45*	1.78***	5.35***
Unknown/missing^c^	61,518	50,065	46,541	103,104
	1	0.81	0.76	1.68
**Deaths**				
Female	15,512	25,328	30,388	96,144
	1	1.63	1.96***	6.20***
Male	17,839	29,099	33,319	115,240
	1	1.63	1.87***	6.46***
Unknown/missing^c^	1,110	677	427	535
	1	0.61	0.38	0.48
Average age-adjusted case rate (× 100,000 people)	748.91	741.87	685.80	631.23
	1	0.99	0.92***	0.84***
Average age-adjusted mortality rate (× 100,000 people)	12.61	13.80	12.36	12.82
	1	1.09**	0.98	1.02
**Vaccination**				
% Completed series	14.20	14.76	14.43	14.17
	1	1.04***	1.02	1.00
% Received 1 dose	21.96	23.44	23.62	24.51
	1	1.07***	1.08***	1.12***

*Data describing trends from January 1, 2020–March 30, 2021.

^a^Comparison of means analyzed using Dunnett's Test (race & ethnicity control = white population; county university enrollment control = none).

^b^The following correspond to the following statistical values: * = *p* < 0.05, ** = *p* < 0.01, *p < 0.001.

^c^Unable to statistically analyse.

**Figure 4:**
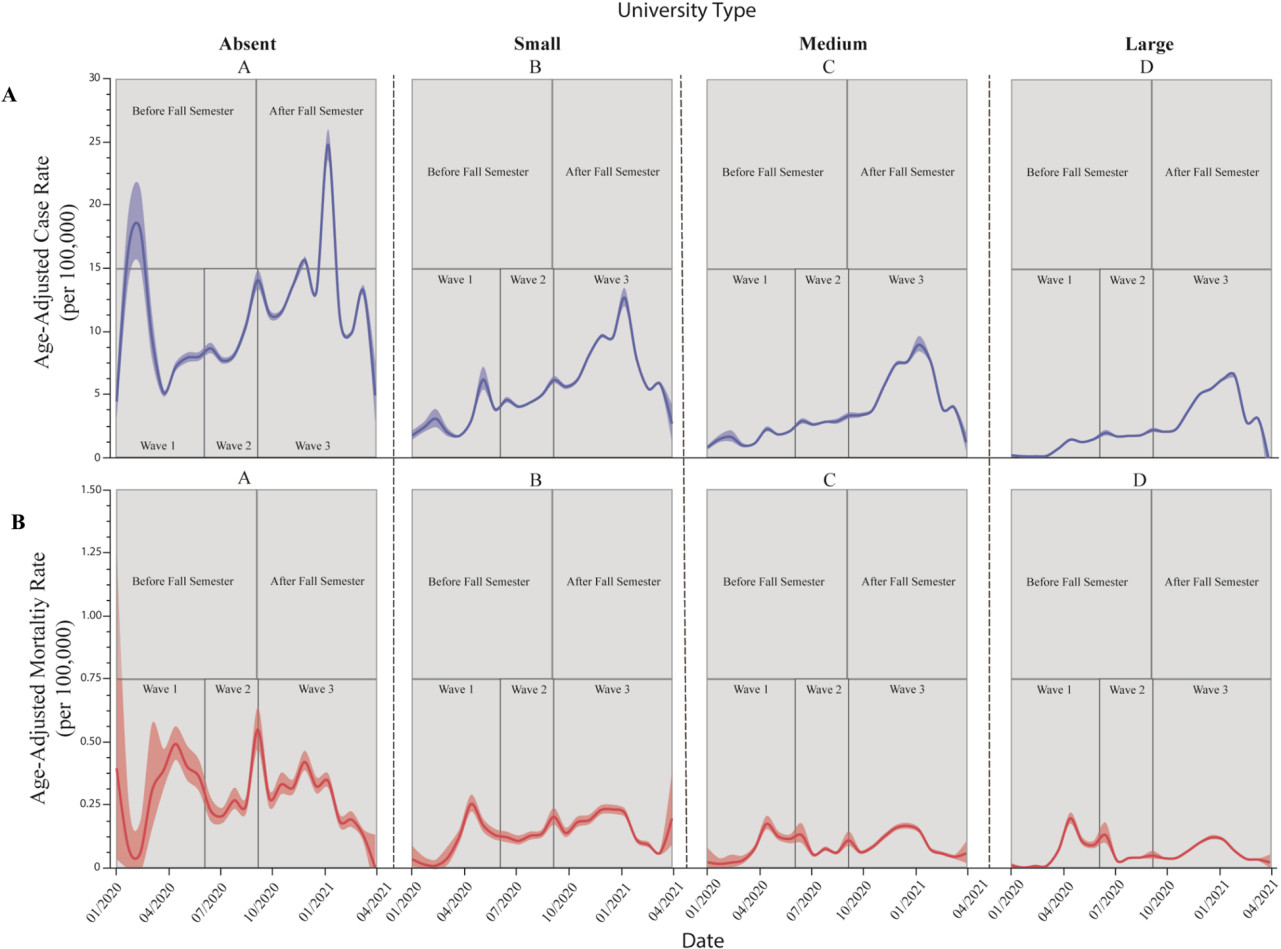
Mean SARS-CoV-2 age-adjusted case (**A**) and mortality (**B**) epidemic curves among United States cases between January 1, 2020 and March 30, 2021. Key periods of interests: before and after Fall 2020 semester begins, and Waves 1–3, are overlaid for visual reference. Shaded widths of each line are 95% confidence limits. Unique letters, arranged in descending order (highest mean value = A and so on), indicate statistically different groups (case and mortality rates, respectively; assessed by Student’s t-test, *p* value $\le$ 0.05).

**Figure 5:**
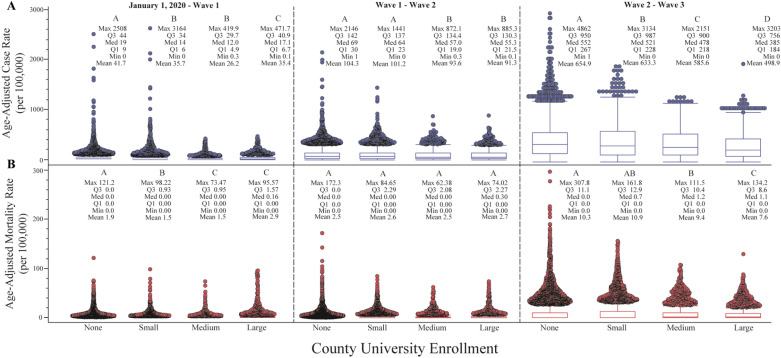
Box plot of mean SARS-CoV-2 age-adjusted case (**A**) and mortality (**B**) rates broken down by each significant outbreak “wave” period. Wave periods were determined by natural breaks in distinctive peaks, based on cases in the United States cases between January 1, 2020 and March 30, 2021. Unique letters, arranged in descending order (highest mean value = A and so on), indicate statistically different groups (case and mortality rates, respectively; assessed by Student’s t-test, *p* value $\le$ 0.05). Individual circles represent outlier counties.

**Figure 6:**
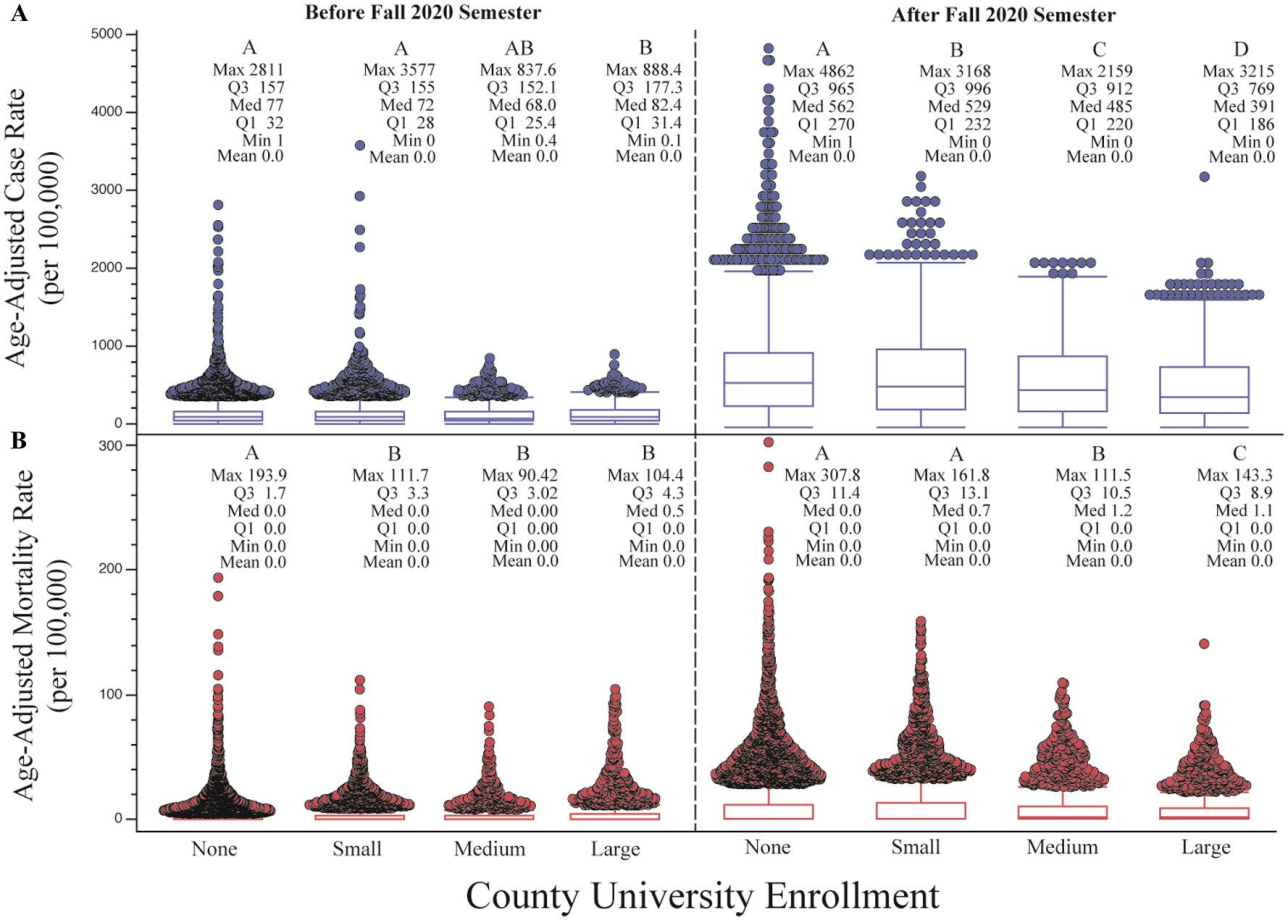
Box plot of mean SARS-CoV-2 age-adjusted case (**A**) and mortality (**B**) rates before and after the Fall 2020 semester period. The fall 2020 semester was assumed to begin September 1, 2020. Unique letters, arranged in descending order (highest mean value = A and so on), indicate statistically different groups (case and mortality rates, respectively; assessed by Student’s t-test, *p* value $\le$ 0.05). Individual circles represent outlier counties.

Across the three wave periods there is a significant reduction in both case and mortality rates with increasing county UE, compared to counties with no university enrollment (Figs. 4 and 5). Case and mortality rates remained below 105 and 2.94 (per 100,000), respectively, for all county types through waves 1 and 2, but markedly increased in wave 3. These rapid increases in both case and mortality rates were experienced across all county types but were less severe as university enrollment increased. The Fall 2020 semester coincided with the beginning of wave 3, and case and mortality rates followed similar trends across all county university types (Figs. 4 and 6).

The effects of each included covariate were similar across county university enrollment types (Table 2). Each of the 18 covariates evaluated in this analysis was included in at least one final best-fit model; variables included in $\ge$ 75% of all models were 2019 population, CCVI, % receiving at least one vaccination dose, and % of time in which childcare facilities, nursing home visitations, and restaurants were closed at the county level. Statistically weighted averages of logWorth values show that enforcing the closure of nursing home visitations and childcare facilities were the two most important covariates for age-adjusted case rates, while 2019 county population & CCVI were the two most important covariates for age-adjusted mortality rates.

**Table 2:**
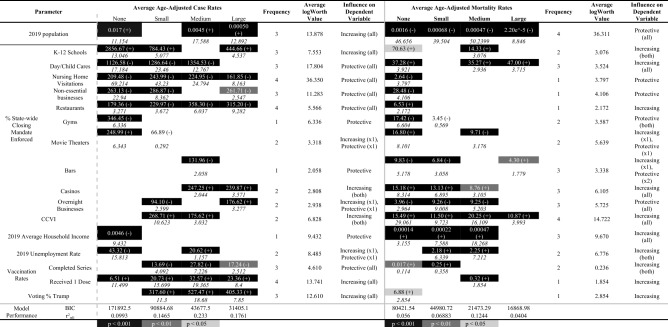
Matrix displaying covariates (n = 18) included in each final model (Aim 1) assessing age-adjusted case (A) and mortality (B) rates by university enrollment and race & ethnicity.

Overall significance for each included covariate is indicated by gray shade (light gray = p < 0.05, dark gray = p < 0.01, black = p < 0.001).

### Factor and cluster analyses (Aim 2)

A total of 1,568 colleges and universities across 740 counties were included in the sub-analysis of college reopening plans for the Fall of 2020 based on availability of reported COVID-19 mitigation plans for the Fall 2020 semester (Table 3). Of these, 235 counties had large enrollments, 196 counties had medium, 302 had small enrollment totals, and seven counties had no reported higher education enrollments.

**Table 3:** Covariates included in (Aim 2) analysis of campus COVID-19 mitigation strategies for Fall 2020 semester (September 1st-December 31st) amongst college/university sub-sample (n = 1568).

Variable	Category		
**Institution category (n = 1568)**			
	Private for-profit (n = 2)		
	Private not-for-profit (n = 994)		
	Public (n = 572)		
**Institution size category (n = 1568)**			
	Under 1000 students (n = 379)		
	1000–4999 (n = 722)		
	5000–9999 (n = 194)		
	10,000–19,999 (n = 156)		
	20,000 and above (n = 117)		
**School land grant status (n = 1568)**			
	Land grant institution (n = 70)		
	Not a land grant institution (n = 1498)		
**School location description (n = 1568)**			
City (n = 698)	City: Large (n = 289)	City: Midsize (n = 159)	City: Small (n = 250)
Rural (n = 126)	Rural: Distant (n = 40)	Rural: Fringe (n = 66)	Rural: Remote (n = 20)
Suburb (n = 398)	Suburb: Large (n = 318)	Suburb: Midsize (n = 50)	Suburb: Small (n = 30)
Town (n = 346)	Town: Distant (n = 183)	Town: Fringe (n = 60)	Town: Remote (n = 103)
**Schools in counties under state mask mandate (n = 1568)**			
	Masks mandated (n = 1159)		
	Masks not mandated (n = 409)		
**Self-reported mask usage frequency (n = 1568)**			
	High (n = 1186)		
	Moderate (n = 308)		
	Low (n = 74)		
**County enrollment (n = 740)**			
	Absent (n = 7)		
	Small (n = 302)		
	Medium (n = 196)		
	Large (n = 235)		
**Mode of instruction for fall 2020 semester (n = 1568)**			
Hybrid (n = 327)	50–50 online and in person (n = 4)		
	Hybrid teaching (n = 63)		
	Professor’s choice (n = 50)		
	Simultaneous teaching (n = 71)		
	Some variety of methods, non-specific plans (n = 139)		
In-person (n = 447)	Fully in-person (n = 58)		
	Primarily in-person, some courses online (n = 389)		
None (n = 7)	Closed (n = 7)		
Online (n = 477)	Already an online institution (n = 9)		
	Fully online, at least some students allowed on campus (n = 42)		
	Fully online, no students on campus (n = 84)		
	Primarily online, some course in person (n = 278)		
	Primarily online, with delayed transition to in-person instruction (n = 64)		
Unknown (n = 310)	Unknown/TBD, Other (n = 275)		
	No COVID mentions (n = 35)		
**COVID-19 testing plans for fall 2020 semester (n = 1568)**			
	Encouraged, not provided or pre-arrival only (n = 115)		
	Mandatory regular testing of all students (n = 88)		
	Mandatory sampling of some students (n = 183)		
	No testing mentioned (n = 769)		
	Only symptomatic testing offered (n = 344)		
	Voluntary testing (n = 69)		

The multivariate factor analysis revealed that the first three dimensions (for which eigenvalues were above one) only explained 20% of the cumulative variance in population adjusted COVID-19 cases during the Fall of 2020 (Table 4). However, within those dimensions, county-level descriptions (i.e. whether there was a state mask mandate, self-reported mask wearing, and county-level enrollment size). contributed 15% more than expected (~ 40% of overall contribution) to the first dimension. The second dimension’s contribution to the variance in population-adjusted COVID-19 cases was strongly defined by institutional factors (~ 50%) (i.e. institutional category, size category, land grant status, and school location description), and campus COVID-19 mitigation strategies (~ 35%) (i.e. MOI category and testing strategy). The third dimension was almost entirely defined by campus COVID-19 mitigation strategies (~ 65% of total contribution). Grouped variables contributing more than expected to these three dimensions (Table 5) were retained for both the cluster analysis and modeling.

**Table 4:** Overall chi-square results for hierarchical cluster analysis factors contributing to county-level population adjusted COVID-19 cases in fall 2020 (September 1st–December 31st).

Factor	*p* value	df
Institution location description	1.92e−156	6
County enrollment size	1.75e−153	6
Institution size	1.06e−136	8
Mask usage category	1.66e−103	6
Institution category	5.54e−89	4
MOI category	4.59e−72	8
Testing type	5.76e−69	10
Land grant	2.77e−19	2
State mask mandate	7.63e−17	2

**Table 5:** Within-cluster (Mod.Cla; percentages of individuals who fit this description are in this cluster) and across-cluster (Cla.Mod; this percentage of individuals in this cluster fit this description) distributions of variables.

Variable	Category	Cluster 1				Cluster 2				Cluster 3			
		Within cluster % (Cla/Mod)	Across cluster % (Mod/Cla)	*p* value	v-test score	Within cluster % (Cla/Mod)	Across cluster % (Mod/Cla)	*p* value	v-test score	Within cluster % (Cla/Mod)	Across cluster % (Mod/Cla)	*P* value	v-test score
Institution type	Private not-for-profit	55.33	89.62	5.39e−71	18.27								
	Private for-profit												
	Public					57.51	70.90	1.80e−74	18.25				
Institution size	Under 1000	62.26	38.57	4.59e−72	10.35								
	1000–4999									43.35	64.27	2.15e−22	9.73
	5000–9999					58.76	24.56	1.39e−19	9.05				
	10,000–19,999					73.71	24.78	2.22e−33	12.03				
	20,000 and above					94.01	23.70	1.91e−53	15.38				
Land grant status	Land grant institution					77.14	11.63	5.39e−17	8.37				
	Not a land grant institution	41.25	100.00	5.76e−69	8.20								
School location	Suburb	69.34	45.05	1.66e−103	14.33								
	Town									79.19	56.26	2.39e−101	21.37
	Rural									70.63	18.27	1.69e−21	9.52
	City	42.55	48.13	2.04e−2	2.31	43.69	65.73	5.27e−28	10.97				
County enrollment	Large	54.86	79.09	2.44e−49	14.50	40.00	77.15	1.77e−26	10.64				
	Medium									45.84	28.33	1.84e−09	6.01
	Small									81.56	59.95	2.38e−117	23.02
	Absent									57.14	1.64	4.82e−02	1.97
On campus testing strategy	No testing mentioned	56.82	70.82	5.54e−89	14.04								
	Encouraged, not provided, or prearrival only									43.47	10.26	3.00e−03	2.90
	Only symptomatic testing offered					50.58	37.50	9.44e−21	9.34				
	Voluntary testing									50.72	7.18	5.40e−04	3.45
	Mandatory sample testing of some students					51.91	20.47	1.62e−11	6.73				
	Mandatory testing of all students					52.27	9.91	5.01e−06	4.56				
MOI	Online					47.37	48.70	1.62e−23	9.99				
	Hybrid					39.44	27.80	1.70e−05	4.30				
	In person									57.49	52.77	2.57e−44	13.96
	Unknown	59.03	29.49	1.28e−53	7.69								
Mask usage	High	49.40	94.32	1.28e−53	14.93	34.23	87.50	1.33e−13	7.40				
	Moderate									71.10	44.96	3.92e−60	16.35
	Low									95.83	14.16	6.11e−33	11.95
State mask mandate status	Masks mandated	43.05	79.57	7.63e−17	4.14	32.35	80.81	4.06e−05	4.10				
	Masks not mandated									47.43	39.83	3.91e−16	8.14

V-test values greater than 1.96 correspond to a *p* values less than 0.05; and indicate an over-represented variable for the category. Only variables with a *p* value < 0.05 are included as this shows that one category is significantly linked to the other categories.

From these factors, we constructed a hierarchical cluster analysis that identified three distinct school clusters (Table 6) that were differentially associated with population-adjusted COVID-19 cases. All factors with a positive value test score above 1.96 (*p* value ≤ 0.05) were retained. Clusters were most heavily defined by the school location setting (e.g. rural vs urban), the county enrollment size, and the size category of the institution. Cluster 1 represented suburban and city-based small private institutions in counties with large student enrollments that did not report COVID measures for the Fall 2020 semester. Cluster 2 represented larger, city-based land grant public institutions that remained online or in hybrid instruction mode for the fall 2020 semester, and that offered multiple testing options for students. Schools in clusters 1 and 2 were in counties under mask mandates and that reported high mask usage. The third cluster was defined by small schools in counties with either no or small student enrollments, that held in-person instruction during the Fall of 2020, offered voluntary or no testing options for students, and whose counties reported low to moderate mask usage and had no mask mandates in place at the state level.

**Table 6:** Best overall fit model that predicts population-adjusted county-level COVID-19 cases during the fall of 2020 (September 1st–December 31st) including college/university characteristics and COVID-19 mitigation strategies. Increasing number of asterisks correspond to the increasingly significant statistical values (e.g. * = p < 0.05, ** = p < 0.01, *** = p < 0.001).

Variable	Coefficient	Standard error	z-value	*p* value
Intercept/Reference	1.59e+00	3.98e−01	3.996	6.44e−05***
**School size**				
10,000–19,999	1.26e−01	5.00e−02	2.522	0.01167*
20,000 and above	3.96e−01	6.03e−02	6.564	5.25e−11***
5000–9999	9.98e−02	4.49e−02	2.224	0.02616*
Under 1,000	5.37e−02	3.39e−02	1.582	0.11365
**School category**				
Private, not-for-profit	− 6.186e−01	3.560e−01	− 1.737	0.08231
Public	− 7.05e−01	3.57e−01	− 1.973	0.04850*
**Land grant status**				
Not a Land Grant Institution	1.56e + 00	6.63e−02	2.352	0.01868*
**School location**				
Rural	1.16e−01	5.46e−02	− 2.114	0.03449*
Suburb	1.16e−02	3.39e−02	0.342	0.73264
Town	− 8.37e−02	4.14e−02	− 2.02	0.04342*
**MOI**				
Online	− 5.56e−02	3.71e−02	− 1.5	0.13367
None (School Closed)	− 1.16e−02	1.94e−01	− 0.599	0.54894
In person	2.26e−02	3.75e−02	0.604	0.54597
Unknown	2.53e−02	4.03e−02	0.628	0.52975
**On-campus testing**				
Mandatory regular testing of all students	− 1.50e−01	7.27e−02	− 2.067	0.03876 *
Mandatory sample testing of some students	− 1.47e−01	6.09e−02	− 2.418	0.01561 *
No testing mentioned	− 4.55e−02	5.14e−02	− 0.885	0.37641
Only symptomatic testing offered	6.11e−03	5.47e−02	0.112	0.91097
Voluntary testing	7.71e−03	7.74e−02	0.1	0.92067
**State mask mandate**				
Masks not mandated	3.62e−01	3.32e−02	10.9	< 2e−16***
**Mask usage**				
Low	3.72e−01	6.52e−02	5.697	1.22e−08***
Moderate	1.94e−01	3.66e−02	5.304	1.13e−07***
**County enrollment**				
Small	− 1.57e−01	1.40e−01	− 1.121	0.2623
Medium	− 5.08e−02	1.40e−01	− 0.362	0.71749
Large	2.81e−02	1.39e−01	0.202	0.8402
**Median household income**	1.07e−05	1.54e−06	6.96	3.40e−12***

### Modeling COVID-19 mitigation plans impact on county-level cases (Aim 2)

Best fit models that examined the association of campus COVID-19 mitigation strategies (MOI, testing strategies, school-specific variables) and economic/county-level covariates (mask usage, mask mandates, median household income) with population-adjusted cases in the Fall of 2020 showed that the most strongly contributive factors to increased county population-adjusted case numbers were low and moderate mask usage, lack of state mask mandates, and median household income (Table 6). For the school-related covariates alone, the overall best fit model predicted that larger schools, schools located in rural areas, and non-land-grant institutions were associated with more county-level cases, (i.e. schools that were part of Cluster 2 were more likely to be located in counties with lower population-adjusted COVID-19 case rates, while those in Clusters 1 and 3 were associated with higher county-level cases). Overall, MOI and testing strategies were not significantly predictive of county-level case rates during the Fall 2020 semester, except that schools that employed mandatory testing of students were associated with counties with lower population adjusted case numbers.

## Discussion

It had been speculated that counties with large university enrollments were at higher risk for COVID outbreaks in the U.S. In this analysis, we evaluated a total of 22,385,335 cases reported to the CDC, representing 3,047 U.S. counties from January 1, 2020 through March 30, 2021. After all cases and deaths were aggregated to the county level and categorized by their total university enrollment sizes, this analysis found that the presence of large university enrollments was associated with lower county COVID-19 case rates.

A retrospective analysis evaluating 15 months of cases and deaths caused by COVID-19 reveals small, but significant reductions in cases among counties with increasing university enrollments. However, little to no change was noted with respect to mortality rates. Further analyses focused on differences between age groups also reveal little to no variation in the case and mortality rates of each group across university enrollment size. However, notable age-related trends were discovered. First, the highest case rates were among young to middle-aged adults (20–59), with 20–29-year-olds experiencing the highest case rates than any other age group—a finding also found by Monod et al.^30^ Second, although the 0–9 and 80+ year old age groups experienced the lowest case rates, the 80+ year old age group’s risk of death was substantially higher than all other age groups. These trends corroborate the widely observed findings pertaining to COVID-19 fatality^31,32^.

The time series plots of daily aggregated county averages of case and mortality rates (Fig. 4) show observable differences in the magnitude of peaks with increasing county university enrollment. However, when evaluating COVID transmission by wave, critical differences become more apparent. Three COVID-19 waves occurred prior to May 2021, with each wave more severe than the wave before. However, the third wave saw a ~ 3-6-fold increase in cases and deaths as compared to waves 1 and 2. Although all counties experienced this drastic increase in COVID-19, those with more university enrollment had significantly lower case rates (5.3, 10.6, and 27.2% for counties with small, medium, and large university enrollments, respectively) compared to counties with no university enrollment. Counties with medium or large university enrollments experienced significantly lower COVID-19 death rates (averaging 12.8 and 29.8% lower, respectively) compared to counties with low or no university enrollments.

The second epidemiologic period of interest was among counties before and after the Fall 2020 semester. As seen in the wave analysis, all counties experienced a rapid increase in both COVID-19 cases and deaths after the start of the Fall 2020 semester. However, counties with increasing university enrollment experienced significantly lower case rates (3.7, 10.8, and 27.1% lower for counties with small, medium, and large university enrollments, respectively) compared to counties with no university enrollment. Counties with medium and large university enrollments also resulted in significantly lower death rates (averaging 13.2 and 30.2% lower, respectively) compared to counties with low or no university enrollment.

Our sub-group analysis of COVID-19 mitigation strategies for the Fall 2020 semester provide further evidence that colleges and universities were not associated with increases in county-level cases—even before cohesive containment plans were established. The cluster analysis identified that schools identified in Cluster 2 tended to be larger, land grant universities that maintained entirely online or hybrid course instruction during the Fall 2020 semester and were likely to be in counties with lower population-adjusted COVID cases. These schools were more likely to have mandatory student testing that was significantly associated with overall lower county cases numbers. During the Fall 2020 semester, county and state-level factors (e.g., mask usage, mask mandates, and median household income) were far more significantly predictive of overall county-level cases, which held true during the larger analysis period.

Overall, the COVID-19 pandemic through March 30, 2021 rapidly spread through all U.S. counties with similar patterns in the timing and intensity of cases and deaths, regardless of the size of university enrollment. However, the magnitude by which cases and deaths affected counties is strongly associated with university enrollment. Although there were minimal differences among death rates by university enrollment, large enrollment universities were most affected by COVID-19 in the early stages of the pandemic—a suspected driving force behind the lack of significant differences in overall mortality rates. However, as the pandemic progressed in intensity (case and deaths per day), counties with increasing university enrollments experienced decreased risks in acquiring, and dying from, COVID-19.

Together these two analyses strongly suggest that community-level variables—and not universities—are what drove COVID-19 cases during this time period. Despite having larger populations, counties with large university enrollments fared better than counties with little or no university enrollments, especially as COVID-19 cases surged through the winter of 2020–2021 (wave 3). In comparison to counties with little or no university enrollment, large university enrollment counties contained higher household incomes, less unemployment, and had higher vaccination rates (% with at least 1 dose). These counties also tended to enforce statewide mandates more frequently and for longer throughout the pandemic compared to counties with little to no university enrollments (Tables S1 and S2).

In addition to the differences noted above, public health decisions were dependent on several political, economic, and social factors. It is apparent that this pandemic has fueled divisions along political lines, which influenced both public health decisions and compliance. From adherence to social distancing and mask wearing to vaccination rates, political associations appear to be a strong influencing force^33^. Using the 2020 U.S. Presidential election as a proxy for determining a county’s political affiliation, the associations of COVID-19 cases and deaths are moderately correlated (Figs. S1 and S2). Counties with higher university enrollments are also correlated with increased overall education rates, which have been found to be associated with use of pandemic control^34–36^.

To date, several studies have analyzed the risk of transmission associated with universities and/or college-aged populations, but all have been limited to no more than 4–5 institutions^2,7,37,38^. Furthermore, very few studies have estimated the attributable risk between universities and students within the communities they reside^39^. Studies that did address community transmission and associations with students were systematic reviews, mathematical simulations, and/or consisted of elementary or primary student ages^10,40,41^. Our retrospective analysis is novel in that all data collected is specific to our central question, narrowing the scope of our investigation to produce tangible estimates of transmission risk at high spatial and temporal scales.

This study is limited in its analysis by aggregating non-lineage-specific individual-level COVID-19 case data to county of residence. By doing so, generalizations are made across varying population sizes and COVID-19 strains and cannot capture subtle differences. Numerous reporting agencies submit varying levels of completeness to the CDC, leading to a high potential for ascertainment bias. Additionally, due to the rapid onset of cases, particularly through peak wave periods, significant delays in reporting also occurred. This analysis attempted to establish etiologic-specific onset dates given the data made available. In some cases, differences of 1 + week from reported onset date to actual, true onset date likely exist and are unavoidable. However, the data account for a significant portion of the U.S. population, thus reducing the errors in generalization and representing the best source for such a study. Limitations to the sub-group analysis included an incomplete picture of mitigation strategies for some of the universities (e.g. Cluster 1 was defined by schools that did not fully report their COVID-19 plans). Counties without higher-education enrollments were also severely limited in this dataset, which could have impacted the analysis. We also assumed the mitigation plans to be static over the course of the Fall 2020 semester due to the lack of available data of changes. Given that this was the first semester with some schools in person, we felt it was likely that a university would maintain proposed plans, and if changes occurred, they would be to bolster mitigation, not reduce.

This study incorporates key differences among American counties stratifying across a spectrum of enrolled university students. In general, counties with increasing enrollment populations tend to be more populated and urban. As such, several potentially important factors associated with COVID transmission, reporting, and knowledge, attitudes, and practices have been generalized or overlooked. Future studies would provide a great service to public health by expanding on the methodologies and results of this study on several of these key factors. For example, counties with large university enrollments appear to have many key protective factors in place to mitigate COVID-19. However, these counties also contain significantly larger populations of black, indigenous, and people of color (BIPOC) that suffer from disproportionate racial and health inequities. Consequently, social vulnerabilities are significantly higher in counties with large university enrollments. Preliminary analyses of this dataset, in respect to COVID-19 outcomes by race and ethnicity, suggest corroborative evidence of these inequities (data not shown). Future studies should also evaluate the co-evolution and/or competition between COVID-19 and other respiratory viruses, particularly seasonal influenza and respiratory syncytial virus conducted in a similar longitudinal analyses as this study.

## Conclusions

1. An increase in a county’s university enrollment is associated with a lower rate of COVID-19 cases and deaths compared to counties with no university enrollments.
2. Counties with the largest universities tend to have greater population sizes, higher household income, lower unemployment, a higher percentage of people partially vaccinated against COVID-19, and a higher proportion of the population voting for Biden in the 2020 presidential election, but also a higher social vulnerability, than those with no university enrollment.
3. Counties with university enrollments tend to have higher enforcement of statewide closing mandates than those without.
4. Despite having high population densities and high social vulnerabilities, individuals in counties with large university enrollments had reduced COVID-19 case and mortality rates than those without university enrollments, especially in the third wave of the pandemic.
5. Increasing education levels, enforcement of closing mandates, adherence to public health recommendations, and political affiliation were all associated with lower COVID-19 case and mortality rates.
6. There was no significant impact on community cases from universities who returned for the Fall 2020 semester; county-level factors were the leading predictors of cases.
7. These results offer evidence against the presumption that universities increase risk of COVID-19 community transmission.

## Supplementary Information

Supplementary Information.

## Data Availability

All data used in this analysis is free and available for public use. Individuals interested in COVID-19 case data can apply and request access via https://data.cdc.gov/.
