# Supplementary material for: Modeling community COVID-19 transmission risk associated with U.S. universities
Source: Sci Rep. 2023 Jan 25;13:1428. doi: 10.1038/s41598-023-28212-z (PMC9875777; doi:10.1038/s41598-023-28212-z)
Supplement: Supplementary file 1 — Supplementary Information. [file 41598_2023_28212_MOESM1_ESM.docx]

| **Type of Statewide Mandate** | **Wave Period** | **County University Enrollment** | | | |
| --- | --- | --- | --- | --- | --- |
|  |  | *Absent* | *Small* | *Medium* | *Large* |
| K-12 Public Schools | 1 | 0.98 | 0.98 | 0.97 | 0.94 |
|  | 2 | 1.00 | 1.00 | 1.00 | 1.00 |
|  | 3 | 1.00 | 1.00 | 0.99 | 1.00 |
| Day/Child Cares | 1 | 0.15 | 0.20 | 0.25 | 0.24 |
|  | 2 | 0.00 | 0.00 | 0.01 | 0.01 |
|  | 3 | 0.00 | 0.00 | 0.00 | 0.00 |
| Nursing Home Visitations | 1 | 0.63 | 0.71 | 0.67 | 0.59 |
|  | 2 | 0.63 | 0.74 | 0.65 | 0.63 |
|  | 3 | 0.52 | 0.69 | 0.63 | 0.63 |
| Non-Essential Businesses | 1 | 0.45 | 0.44 | 0.53 | 0.53 |
|  | 2 | 0.01 | 0.00 | 0.01 | 0.01 |
|  | 3 | 0.02 | 0.01 | 0.01 | 0.00 |
| Restaurants | 1 | 0.55 | 0.55 | 0.65 | 0.65 |
|  | 2 | 0.01 | 0.02 | 0.04 | 0.11 |
|  | 3 | 0.10 | 0.09 | 0.12 | 0.19 |
| Gyms | 1 | 0.64 | 0.66 | 0.74 | 0.75 |
|  | 2 | 0.07 | 0.12 | 0.20 | 0.34 |
|  | 3 | 0.04 | 0.04 | 0.08 | 0.17 |
| Movie Theaters | 1 | 0.73 | 0.75 | 0.78 | 0.78 |
|  | 2 | 0.13 | 0.21 | 0.31 | 0.41 |
|  | 3 | 0.10 | 0.12 | 0.18 | 0.28 |
| Bars | 1 | 0.73 | 0.64 | 0.74 | 0.77 |
|  | 2 | 0.11 | 0.13 | 0.20 | 0.44 |
|  | 3 | 0.16 | 0.20 | 0.29 | 0.43 |
| Casinos | 1 | 0.34 | 0.32 | 0.41 | 0.40 |
|  | 2 | 0.03 | 0.04 | 0.07 | 0.15 |
|  | 3 | 0.06 | 0.06 | 0.05 | 0.07 |
| Overnight Businesses | 1 | 0.07 | 0.08 | 0.02 | 0.03 |
|  | 2 | 0.04 | 0.04 | 0.03 | 0.02 |
|  | 3 | 0.22 | 0.25 | 0.23 | 0.21 |

**Supplemental Table 1.** Percentage of counties enforcing statewide mandates of interest (n=10) by total university enrollment, at any point during each of the three peak wave periods of the COVID-19 Pandemic in the United States between January 1, 2020 and March 30, 2021. Wave periods dates are overlaid continuous case and mortality rates by county university enrollment in Figure 4.

| **Type of Statewide Mandate** | **Before or After Fall semester began** | **County University Enrollment** | | | |
| --- | --- | --- | --- | --- | --- |
|  |  | *Absent* | *Small* | *Medium* | *Large* |
| K-12 Public Schools | 0 | 1.00 | 1.00 | 0.99 | 0.98 |
|  | 1 | 1.00 | 1.00 | 0.99 | 1.00 |
| Day/Child Cares | 0 | 0.04 | 0.06 | 0.09 | 0.10 |
|  | 1 | 0.00 | 0.00 | 0.00 | 0.00 |
| Nursing Home Visitations | 0 | 0.64 | 0.74 | 0.66 | 0.61 |
|  | 1 | 0.52 | 0.68 | 0.63 | 0.63 |
| Non-Essential Businesses | 0 | 0.12 | 0.13 | 0.18 | 0.22 |
|  | 1 | 0.02 | 0.01 | 0.01 | 0.00 |
| Restaurants | 0 | 0.15 | 0.17 | 0.23 | 0.33 |
|  | 1 | 0.10 | 0.09 | 0.12 | 0.19 |
| Gyms | 0 | 0.22 | 0.27 | 0.38 | 0.51 |
|  | 1 | 0.04 | 0.04 | 0.08 | 0.17 |
| Movie Theaters | 0 | 0.29 | 0.36 | 0.47 | 0.56 |
|  | 1 | 0.10 | 0.12 | 0.18 | 0.28 |
| Bars | 0 | 0.27 | 0.28 | 0.38 | 0.57 |
|  | 1 | 0.16 | 0.20 | 0.29 | 0.43 |
| Casinos | 0 | 0.11 | 0.12 | 0.19 | 0.25 |
|  | 1 | 0.06 | 0.06 | 0.05 | 0.07 |
| Overnight Businesses | 0 | 0.05 | 0.05 | 0.02 | 0.03 |
|  | 1 | 0.21 | 0.25 | 0.23 | 0.20 |

**Supplemental Table 2.** Percentage of counties enforcing statewide mandates of interest (n=10) by total university enrollment, at any point before or after the Fall 2020 Academic Semester. Fall 2020 Semester dates are overlaid continuous case and mortality rates by county university enrollment in Figure 4.

**Supplemental Methods.**

*Other Key Covariates*

The CCVI identifies the vulnerability of communities to COVID-19 by aggregating dozens of variables across six themes. This index, specific to COVID-19, provides a powerful insight into individual counties’ unique characteristics, providing a robust and specific assessment of vulnerability. U.S. Presidential Election results were included in this analysis due to the observed relationship between pandemic response and political affiliation (Gao and Radford 2021; Roberson 2021).

**Supplemental Figure Legends**

**Supplemental Figure 1.** Association of population size and total university enrollment for each county in the United States. Each dot represents a county or U.S. County equivalent (including U.S. Territories, n = 3,152) and is color-coded and sized according to mean age-adjusted case rate and % Trump vote in the 2020 U.S. presidential election, respectively. Case rates are adjusted by quartiles where red indicates highest cases and blue indicates lowest cases, relative to the U.S. case rate average. The shaded on the best-fit line indicate 95% confidence limits of the mean.

**Supplemental Figure 2.** Association of population size and total university enrollment for each county in the United States. Each dot represents a county or U.S. County equivalent (including U.S. Territories, n = 3,152) and is color-coded and sized according to mean age-adjusted mortality rate and % Trump vote in the 2020 U.S. presidential election, respectively. Case rates are adjusted by quartiles where red indicates highest deaths and blue indicates lowest deaths, relative to the U.S. case rate average. The shaded on the best-fit line indicate 95% confidence limits of the mean.


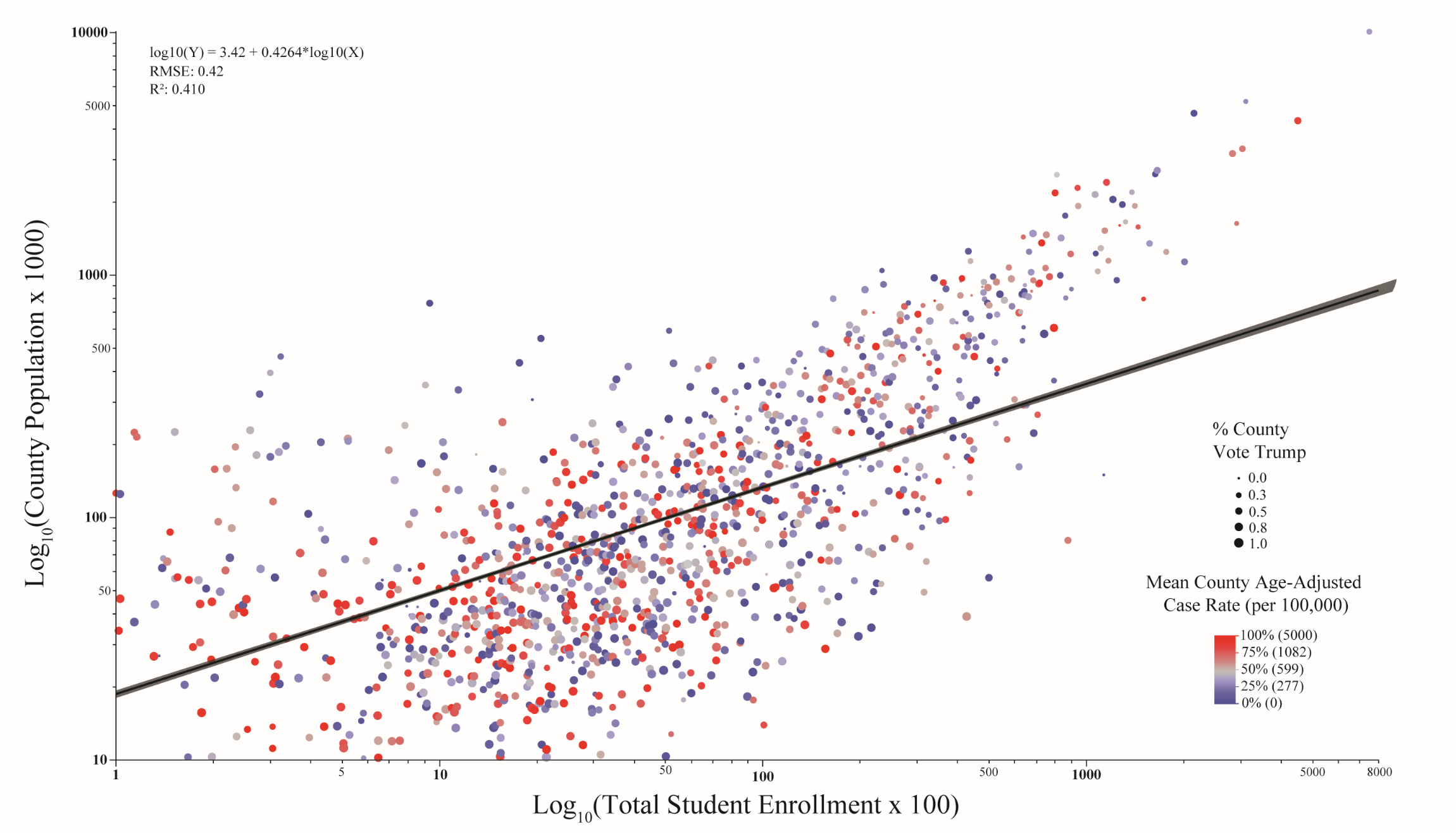


**Supplemental Figure 1.**


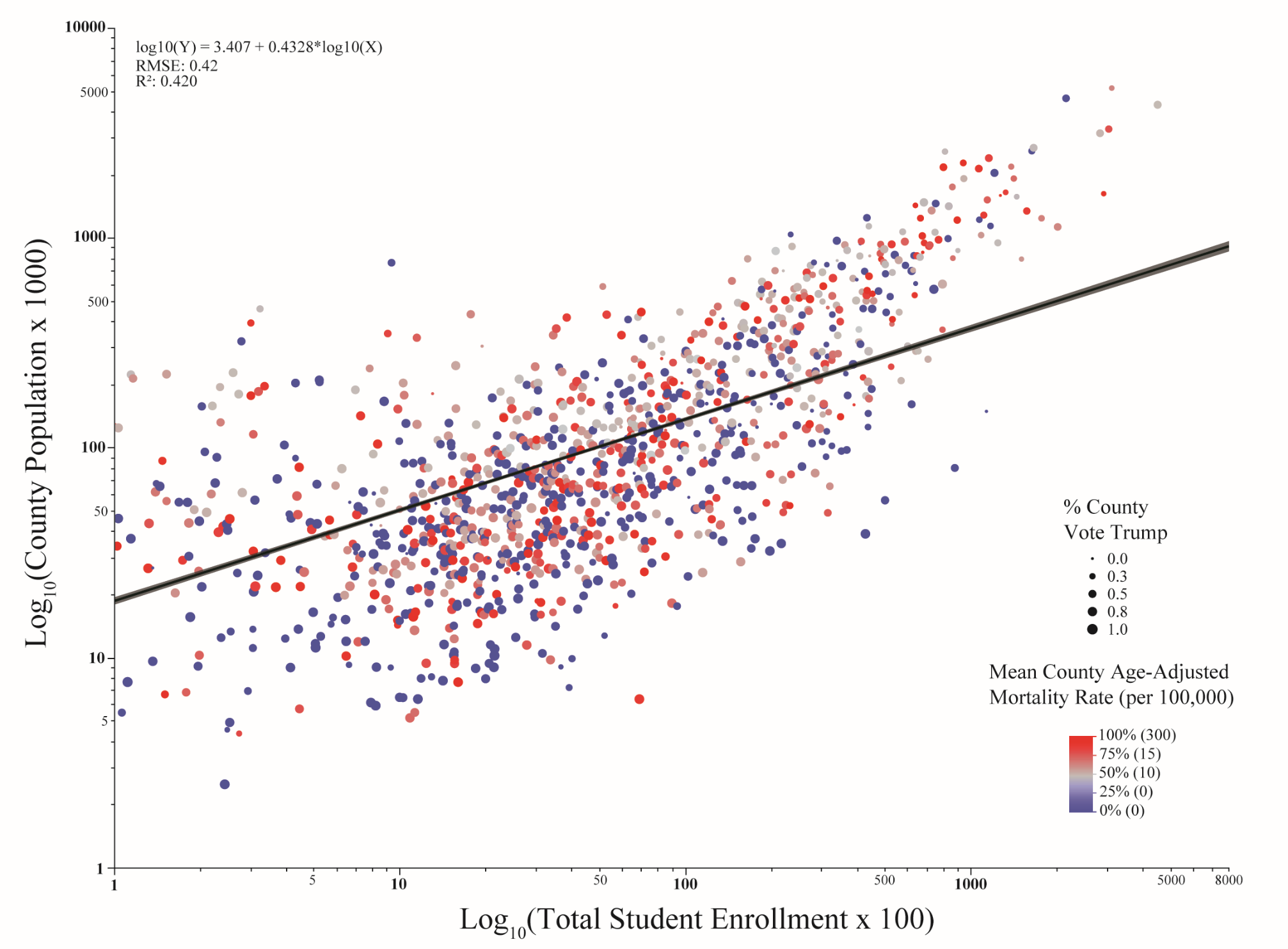


**Supplemental Figure 2.**
